# SNP'ing for longevity

**DOI:** 10.18632/aging.100048

**Published:** 2009-05-06

**Authors:** Jan Vijg

**Affiliations:** Albert Einstein College of Medicine, Bronx, NY 10461, USA

**Keywords:** single nucleotide polymorphism, centenarians, aging, p21, genome instability

Medieval alchemists, craving the boon of youth and desiring renewed health and strength, were kept busy searching for the mythical elixir of life, a universal medicine supposedly containing a recipe for rejuvenescence. Now, in the 21^st^ century, their modern successors use the fruits of the genomics revolution to discover genetic factors that ward off aging and aging-related diseases. In this issue of Aging, Gravina et al. [1]  present evidence that the long life span of the more fortunate among us could be related to variants of the p21 cell cycle inhibitor.

Longevity is simply defined as the property of being long-lived, i.e., approaching the life span of the oldest individual of a species or a population. The latter is also called ‘maximum life span', which in contrast to life expectancy (mean age at death in a population) is not greatly influenced by environmental conditions. Longevity is attained by keeping aging at bay. Aging can be defined operationally as a time-dependent loss of fitness that begins to manifest after the organism attains its maximum reproductive competence. Unlike longevity, this loss of fitness cannot easily be caught in some simple measurement. There are no real biomarkers for aging and its phenotype is extremely complex.

The large variation in maximum life span among species points toward genetic factors that specify the mechanisms that protect against aging and disease. The identification of these genetic factors is of interest since they would provide targets for modes of prevention and intervention, eventually offering everybody a long and healthy life. In short-lived, model organisms such as worms and flies, multiple loci have been demonstrated to affect longevity as a genetic trait. Indeed, specific mutations in genes participating in pathways that are involved in growth and reproduction were shown to confer significant extension of the natural life span of these species [2]. Unfortunately, since little is known about the aging phenotypes that emerge over time to inexorably limits longevity in these species it has been difficult to extrapolate these results to humans. Hence, new model systems need to be found that provide more immediate links to human longevity and healthy aging. Thus far, humans themselves have provided the best such model system.

With the passing of the years, genetic factors play an increasingly important role as determinants of lifespan. At the extreme end of this spectrum we find centenarians, people who attained the age of 100 years or more. This is unusual because current life expectancies are in the range of 70-80, at best. Centenarians escape the most common age-related diseases, which may be why they are exceptionally long-lived individuals [3]. However, it is also possible that they avoid or delay disease because of an inherently slower rate of intrinsic aging. In particular, cancer is absent or significantly delayed in centenarians and it is not at all unlikely that genetic variation at the loci that control tumorigenesis distinguish centenarians from their less fortunate brethren. Gravina et al. [1] put this hypothesis to the test by genotyping a population of Italian centenarians and a younger, control group for single nucleotide variation in the p21 gene [1]. The results revealed at least two rare variants, one in the coding regions leading to an amino acid substitution and another in the 3'-untranslated region, that were significantly under-represented among the centenarians. As argued by the authors, these p21 alleles may be detrimental to longevity and therefore negatively selected in centenarians. The question remains, how do these variants affect p21 functioning and what is the role if any of p21 in determining human life span? The authors consider the possibility that the identified rare alleles increase cancer risk and that their absence would increase the chance to live a cancer-free and therefore long life. This is plausible, but other scenarios are possible.

**Figure 1.**
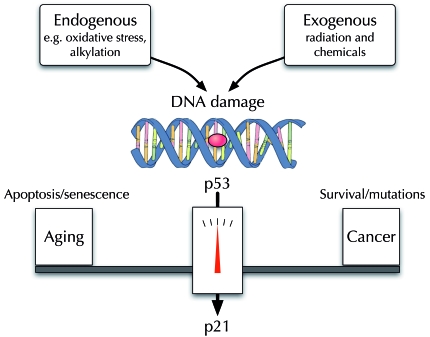
SNP variants in the p21 gene may attenuate the pro-aging activities of p53 without increasing genome instability and cancer.

The cyclin-dependent kinase inhibitor p21^WAF1/CIP1^ is oneof the downstream targets of p53, the so-called guardian of the genome [4]. However, p21 is not as critical a tumor suppressor as p53. Unlike p53, few, if any, p21 mutations have been found in human tumors and p21 knockout mice do not develop many more spontaneous malignancies than their wildtype littermates [5]. Interestingly, evidence has been found that loss of p21 enhances survival of mice with a telomere dysfunction [6]. Hence, p21 may be a promoter of aging, and as such possibly the effector of the pro-aging characteristics of p53 [7]. Constitutive activation of p53 has been demonstrated as a likely cause of premature aging in a number of different mouse models, including animals expressing truncated p53 isoforms [8]. It is possible that one could retain tumor suppressing capability through p53 while simultaneously opposing its pro-aging activity through inhibition of p21 (Figure 1).

Based on the above, it is conceivable that variations in p21 may affect its dual role in aging and cancer, shifting the balance to its pro-aging capacity while retaining robust tumor suppression. If followed up by functional studies, the SNP variants discovered by Gravina et al. could be an important step on our way to developing interventions to blunt p53's pro-aging actions while retaining its anti-aging effects.
